# Surgical repair of isolated right pulmonary artery using pulmonary artery flap and preserved PDA stent

**DOI:** 10.1186/s13019-026-03944-x

**Published:** 2026-03-10

**Authors:** Fumiya Yoneyama, E. Oliver Aregullin, Javier Brenes

**Affiliations:** 1 Congenital Heart Surgery, Texas Children’s Hospital, Austin, TX USA; 2 Pediatric Cardiology, Texas Children’s Hospital, Austin, TX USA; 3 Congenital Heart Surgery, Texas Children’s Hospital, Baylor College of Medicine, TX 6651 Main St, Houston, 77030 USA

## Abstract

Isolated right pulmonary artery (RPA) from the ductus arteriosus is rare and often managed in stages to promote vessel growth. We present a 12-year-old girl with ductal-origin RPA who had undergone patent ductus arteriosus (PDA) stenting at age three and serial balloon dilations. Through median sternotomy, a posterior wall flap was created from the main pulmonary artery (MPA) and rotated toward the RPA, incorporating the preserved stented PDA segment as the posterior wall. The anterior wall was reconstructed with a decellularized pulmonary homograft patch, creating a 9 mm lumen. The patient recovered uneventfully, and postoperative imaging confirmed a widely patent RPA without a gradient. Preserving the PDA stent and combining it with an MPA flap and homograft augmentation offered a durable repair for long-gap ductal-origin RPA, avoiding synthetic conduit use and minimizing dissection.

**Supplementary Information:**

The online version contains supplementary material available at 10.1186/s13019-026-03944-x.

## Introduction

Isolated right pulmonary artery (RPA) originating from the ductus arteriosus is a rare congenital anomaly. The native RPA is often diminutive or disconnected, necessitating staged interventions to encourage growth prior to definitive repair [1, 2]. Ductal stenting has become a valuable strategy to maintain perfusion and promote RPA development, typically followed by surgical unifocalization using interposition grafts or patch-based reconstruction [3, 4]. However, repair becomes particularly challenging when a long anatomical gap exists between the MPA and RPA. We describe a case in which reconstruction was achieved using an MPA flap and homograft patch, preserving the stented ductal segment in situ.

## Case report

**Fig. 1:**
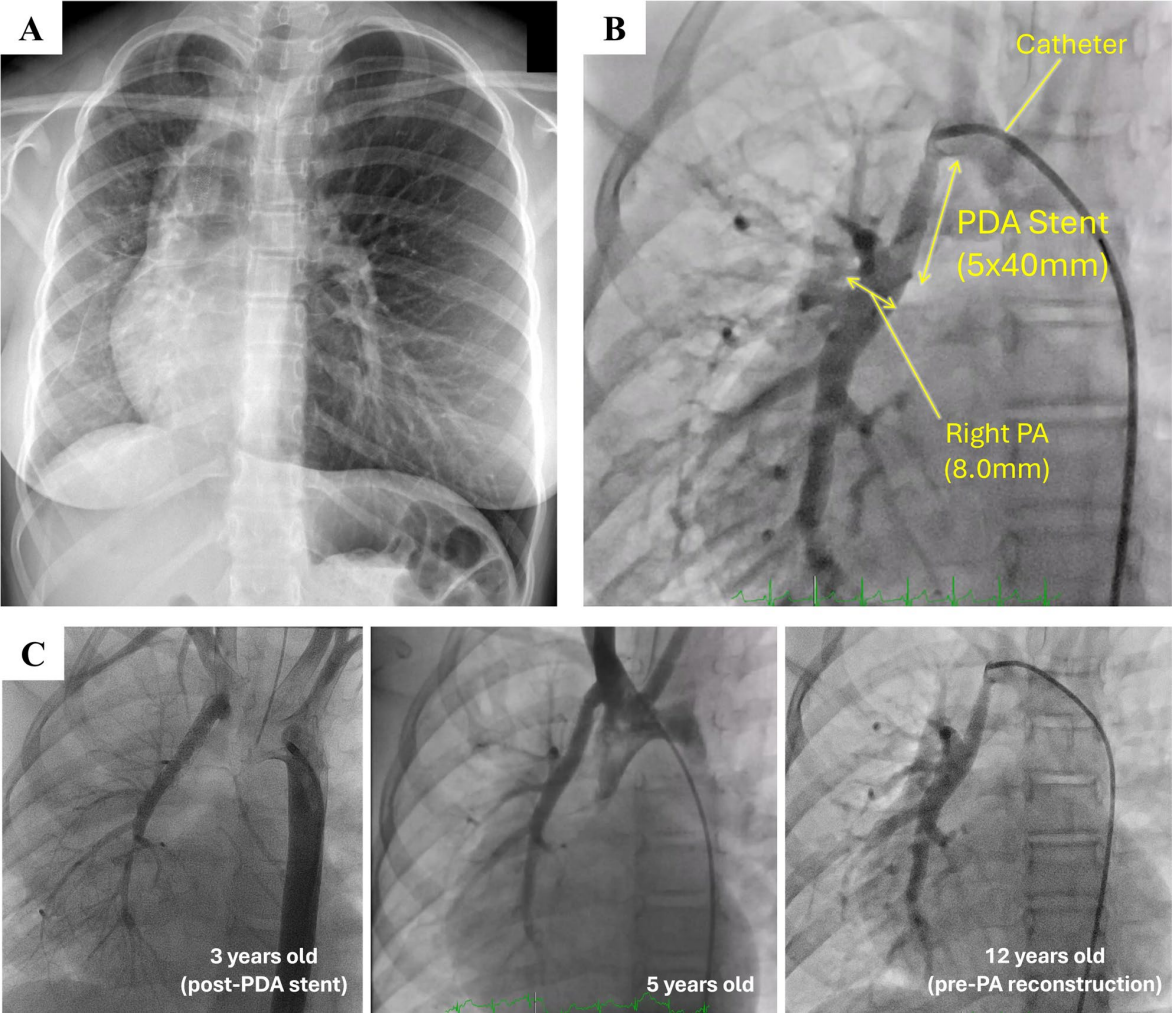
**A**: Preoperative Chest X-ray, **B**: preoperative cardiac catheterization, **C**: catheterization series

A 12-year-old girl (37 kg) with isolated RPA of ductal origin had previously undergone patent ductus arteriosus (PDA) stenting at 3 years of age, followed by serial balloon dilations to promote RPA growth. She maintained low-dose aspirin after PDA stenting. Chest X-ray demonstrated a hypoplastic right lung, and dextroposition (Fig. 1A). Preoperative echocardiography showed normal intracardiac anatomy and preserved biventricular function. Cardiac catheterization revealed a stented, hypoplastic RPA, about 8 mm diameter, with a systolic pressure of 45 mmHg at the distal portion (Fig. 1B). Serial catheter angiography during follow-up demonstrated interval changes in the stented RPA caliber (Fig. 1C).

**Fig. 2:**
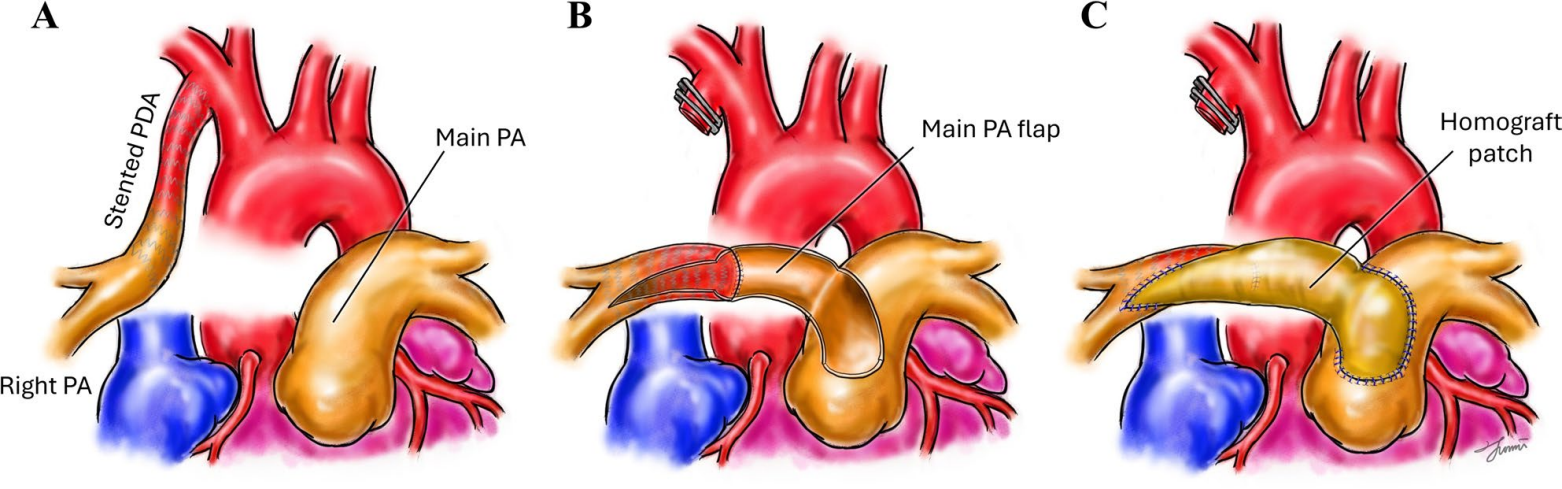
(**A**) Isolated right PA supplied via a stented PDA, (**B**) The stented PDA is divided and opened longitudinally, and a posterior-wall flap is fashioned from the main PA. (**C**) The anterior wall is reconstructed with a pulmonary homograft patch. PA = pulmonary artery, PDA = patent ductus arteriosus

**Fig. 3:**
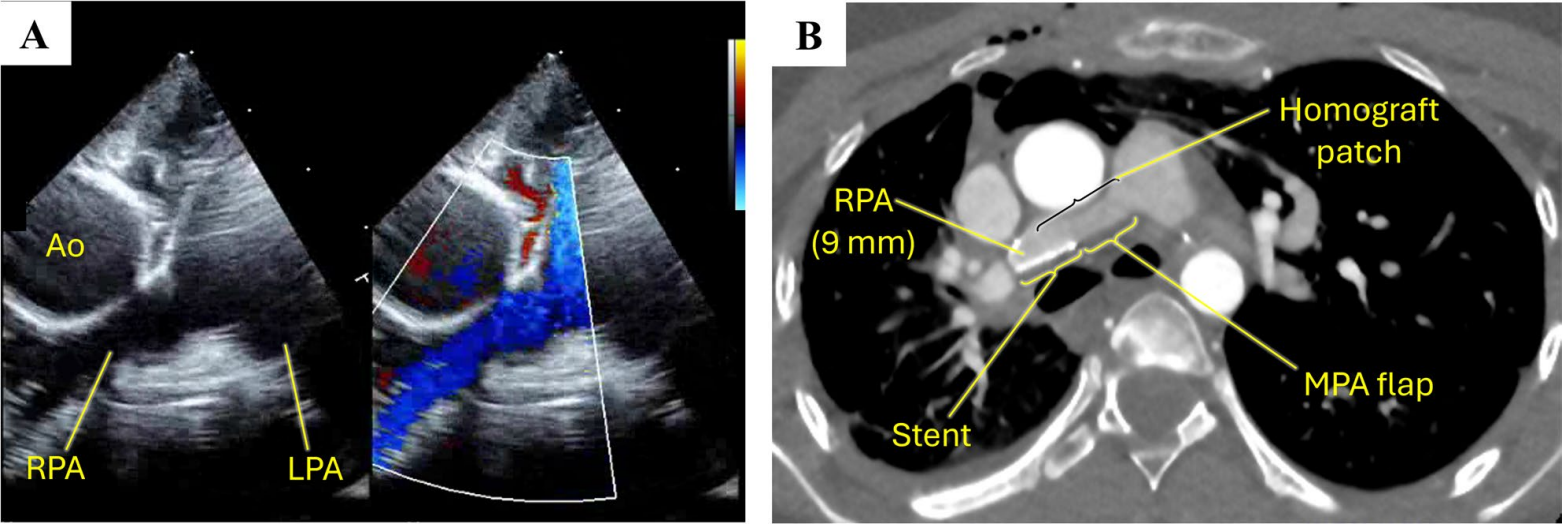
** A**: Postoperative echocardiography for RPA, **B**: postoperative chest computed tomographyA: Postoperative echocardiography for RPA, B: postoperative chest computed tomography Ao = aorta, LPA = left pulmonary artery, MPA = main pulmonary artery, RPA = right pulmonary artery

Through a median sternotomy, cardiopulmonary bypass (CPB) was established, and the patient was cooled to 34 °C (Fig. 2A). The stented PDA was divided at its origin from the innominate artery and opened longitudinally; the opened stented segment was extended distally to just proximal to the RPA bifurcation without trimming or modifying the stent (Video). The gap between the RPA bifurcation and the main pulmonary artery (MPA) measured 30 mm. A generously sized posterior-wall flap was created from the MPA and rotated beneath the ascending aorta toward the opened stented segment, forming the posterior wall of the reconstructed RPA (Fig. 2B). The anterior wall was then reconstructed with a decellularized pulmonary homograft patch spanning from the distal RPA to the MPA, completing a composite lumen (posterior wall: MPA flap + opened stented segment; anterior wall: homograft patch) (Fig. 2C). Dissection around the long-standing stented ductal segment was kept as limited as feasible to avoid stent-related trauma. The reconstructed RPA lumen was calibrated to 9 mm (confirmed with passage of a 9-mm Hegar dilator). The patient was weaned from CPB uneventfully. Postoperative epicardial echocardiography demonstrated no obstruction or gradient across the reconstructed RPA (Fig. 3A), and MPA pressure measured less than one-third of systemic pressure. She was extubated in the operating room and recovered without complications. Her low-dose aspirin was continued postoperatively following definitive reconstruction, and postoperative computed tomography showed a widely patent RPA (Fig. 3B). Follow-up echocardiography at postoperative 3 months showed no RPA obstruction with low velocity.

## Discussion

Surgical repair of isolated RPA has been reported using various techniques, including direct anastomosis, interposition grafts using autologous pericardium or synthetic materials, and MPA flaps [3, 4]. Recent multicenter experience underscores that anastomotic/branch stenosis and reintervention remain common after discontinuous pulmonary artery repair, highlighting the importance of techniques that preserve lumen caliber and minimize dissection in challenging long-gap anatomy [5]. Contemporary surgical series also demonstrate frequent use of patch augmentation and occasional interposition graft strategies when direct anastomosis is not feasible after PA rehabilitation, providing a framework to contextualize the present hybrid reconstruction [6]. Among these, the MPA flap technique is advantageous in pediatric patients because it maintains anatomical continuity with autologous tissue and supports future growth, allowing for potential ballooning or stenting if needed [1]. However, the applicability of MPA flapping alone is often limited by anatomical distance.

In ductal-origin isolated RPA, as in the present case, prior PDA stenting can serve a dual purpose: preserving pulmonary blood flow and facilitating vessel growth during early stages, while also providing structural support for later reconstruction. Although primary repair at initial diagnosis may be feasible in select cases, current strategies increasingly favor staged rehabilitation with PDA stents or shunts before definitive repair [2, 7]. In this setting, direct reimplantation/direct anastomosis may be limited by long-gap tension, and conduit interposition introduces non-growing material with potential late mismatch, while MPA flap alone may not provide sufficient coverage to achieve an adequate lumen caliber. Therefore, our hybrid reconstruction—incorporating the opened stented ductal segment into the posterior wall with an MPA flap and completing the anterior wall with homograft patch augmentation—was designed to restore a smoothly contoured lumen while minimizing dissection and avoiding stent removal. This staged approach encourages RPA maturation and results in more stable long-term outcomes.

Our technique leveraged the existing stented PDA segment as the posterior wall of the reconstructed RPA, avoiding stent removal and minimizing dissection. A posterior MPA flap and anterior homograft patch bridged the 30-mm gap without requiring synthetic conduit material. In this case, definitive repair was performed at 12 years of age after confirming sufficient pulmonary artery growth, and the timing was influenced by family-related scheduling and follow-up logistics. Postoperatively, the reconstructed RPA was widely patent with no hemodynamic gradient. This hybrid approach may offer a durable and anatomically favorable solution in cases of long-gap ductal-origin isolated RPA. Because the posterior wall incorporates a long-standing stented ductal segment, its long-term remodeling and growth potential remain uncertain; therefore, meticulous longitudinal follow-up is required, with a low threshold for catheter-based redilation and/or stent optimization if restenosis occurs.

## Supplementary Information

Supplementary Material 1: Video Intraoperative video showing reconstruction of isolated RPA using a posterior MPA flap and anterior homograft patch. The preserved PDA stent was incorporated into the posterior wall.

## Data Availability

No datasets were generated or analysed during the current study.
